# Influence of mood disorders on outcomes of polycystic ovarian syndrome - A national inpatient sample study - 2016–2020

**DOI:** 10.1016/j.cpnec.2025.100305

**Published:** 2025-06-04

**Authors:** Bob Weng, Reid Morrissey, Jenna Lehn, Mustafa Beidas, Tauseef Abubakar, Mohsin Mirza

**Affiliations:** a Creighton University School of Medicine, Omaha, NE, United States of America; b Creighton University Department of Internal Medicine, CHI Health Bergan Mercy Hospital, Omaha, NE, United States of America

**Keywords:** Polycystic ovarian syndrome, Metabolic disorder, Mood disorder, Demographic, Database study

## Abstract

**Background:**

Polycystic ovarian syndrome (PCOS) is a dysregulated metabolic disorder causing hyperandrogenism, oligomenorrhea/anovulation, and ovarian cysts. The effects of PCOS extend beyond the reproductive detriments, notably an association with mood disorders. Existing literature is limited but suggests comorbid mood derangements increase the severity of PCOS symptoms and comprise a significant portion of healthcare costs in the U.S. Our study aims to further examine the impact of PCOS on the hospitalization course and costs.

**Methods:**

Using data for females ages 18–50 from the National Inpatient Sample (NIS) between 2016 and 2020, a PCOS group was compared to a non-PCOS group for women hospitalized with mood disorders. Length of stay (LOS), hospital costs, and demographic characteristics were analyzed.

**Results:**

PCOS patients accumulated significantly higher LOS and hospital cost. They also were significantly younger, predominantly Caucasian, covered by private insurance, and earned more income. There was a disproportionate use of Medicare for both groups given the younger sample.

**Conclusion:**

Further investigation of demographic nuances and a multidisciplinary approach to PCOS, including policy changes and patient education starting at a young age, should be taken to better understand disease impact on different communities and address the broad scope of the disease (i.e. psychosocial) to reduce its healthcare burden.

## Highlights

• Women hospitalized with mood disorders and concomitant PCOS stayed longer and accrued greater hospital costs than those without PCOS.
• With each decade, the difference in hospital costs for women with mood disorders and PCOS also comparatively increased.
• Women with mood disorders and PCOS were wealthier, carried private insurance, and predominantly Caucasian.

## 1 Introduction

Polycystic ovarian syndrome (PCOS) is an endocrinological disorder affecting women characterized by a multitude of physiologic derangements characterized by hyperandrogenism and insulin resistance [1]. Diagnosis is established according to the Rotterdam criteria requiring two of three symptoms: oligo- or anovulation, hyperandrogenism (e.g. hirsutism, acne), and/or presence of polycystic ovaries on pelvic ultrasound [[2], [3], [4]]. Due to the nature of the principal clinical manifestations centered around menstrual dysfunction and subfertility, metabolic syndrome, type 2 diabetes mellitus, hypertension, metabolic dysfunction-associated steatotic liver disease (MASLD), and obstructive sleep apnea (OSA) are physiological comorbidities closely associated with this condition [2,5]. At the population scale, PCOS affects women of reproductive age, with an incidence of 15–20 % of the population at risk6.

Current management strategies for PCOS patients involve a multidisciplinary approach focused on largely symptomatic control. However, this approach to care has not adequately addressed the considerable hindrance to quality of life. In Alur-Gupta, S et al., women with PCOS were disproportionately found to be at an increased risk for anxiety compared to those without PCOS [7]. Rodriguez-Paris, D. et al. demonstrated notable associations to major depressive disorder (MDD), bipolar disorder (BP), generalized anxiety disorder (GAD), multiple personality disorders, social phobias, obsessive-compulsive disorder (OCD), attention deficit hyperactivity disorder (ADHD), and several eating disorders [8]. Hollinrake, E. et al. concluded there was more than a four-time increase in overall risk of depressive disorders in women with PCOS compared to the normal population [9]. Further studies have also established a robustly negative impact on self-esteem, higher rates of depression, and greater body image distortions [1,2,10]. Thus, prior research suggests one of the most substantial consequences of PCOS lies not within the physiological domain but rather the psychosocial, significant incapacitation of mental and emotional health secondary to physical disturbances place patients in long-term vulnerable positions of isolation, suicidality, and socioeconomic disadvantage.

Within the framework of healthcare burden in the United States, the estimated total economic burden of PCOS exceeded $15 billion a year [11]. While there is currently a considerable lack of literature analyzing the total healthcare costs of PCOS in relation to mood disorders, it is evident from the limited analysis available that a significant portion of PCOS-related healthcare costs are involved in mediating its mood disorders. Further investigation is necessary, however, to parse out the nuances of specific mood disorders and their impact on women's hospital course, prognosis, and the associated healthcare burden.

## 2 Methods

The Nationwide Inpatient Sample (NIS) database was utilized to analyze disparities between groups with PCOS and mood disorders from 2016 to 2020. The Nationwide Inpatient Sample is part of the Healthcare Cost and Utilization Project (HCUP) division of databases sponsored by the Agency for Healthcare Research and Quality (AHRQ); when weighted, the NIS represents approximately 144 million discharges from US hospitals. The NIS is a publicly available, fully identified, HIPAA-compliant database. The primary objective of this retrospective analysis was to analyze the length of stay and total healthcare costs as outcomes to compare groups based on race, age, ethnicity, and median household income by zip code.

Data for females between ages 18–50 years old were extracted between 2016 and 2020 from the NIS. Patients with a PCOS diagnosis (ICD 10 code E28.2) and comorbid mood disorders (anxiety, mania, bipolar disorder per ICD-10 codes were compared to patients with mood disorders without an existing PCOS diagnosis (Supplemental Table 1). Length of stay (LOS) and hospital costs were measured as endpoint parameters between the PCOS and non-PCOS groups to generate unadjusted and adjusted rate ratios (RR), accounting for age, insurance, hospital type, weekend versus weekday admission, and income quartile. Multivariable analysis was subsequently performed to sub-stratify patient characteristics based on age, insurance, hospital type, admission time, income, and specific mood disorder. Statistical significance was set at p < 0.05.

## 3 Results

LOS was significantly longer in the PCOS group (4.43 days) than the non-PCOS group (4.08 days), with an unadjusted ratio of 1.09 (95 % CI = 1.05–1.12) (p=<0.001) and an adjusted ratio of 1.11 (95 % CI = 1.07–1.14) (p=<0.001) (Table 1). Hospital costs, per the USD value in 2020, showed significantly greater costs in care for the PCOS group ($4129) than the non-PCOS group ($3826), with an unadjusted ratio of 1.08 (95 % CI = 1.05–1.11) (p=<0.001) and an adjusted ratio of 1.09 (95 % CI = 1.07–1.13) (p=<0.001). Further examination was done for hospital costs by age in decades (Table 2). For women by age 20, the non-PCOS group accrued $3709 on average compared to $3954 by the PCOS group (RR 1.07 [95 % CI 1.02–1.12]). The difference between the two groups increased by age 30, totaling $3971 by non-PCOS women and $4377 by PCOS women (RR 1.10 [95 % CI 1.06–1.14]). There was a further increase by age 40, totaling $4251 in non-PCOS women and $4847 (RR 1.14 [95 % CI 1.09–1.20]).

**Table 1:** RRs for LOS and hospital costs between PCOS and non-PCOS patients hospitalized with mood disorders. RRs were adjusted for age, insurance type, hospital type, admission time, income, and individual mood disorders.

Metric	No PCOS	PCOS	Unadjusted Rate Ratio (RR) (95 % CI)	p-value	Adjusted Rate Ratio (RR) (95 % CI)	p-value
Length of Stay (LOS)	4.08	4.43	1.09 (1.05–1.12)	<0.001	1.11 (1.07–1.14)	<0.001
Avg. Hospital Costs (USD in 2020)	3826	4129	1.08 (1.05–1.11)	<0.001	1.09 (1.07–1.13)	<0.001

**Table 2:** Age-cost interactions between PCOS and non-PCOS patients hospitalized with mood disorders.

Age (years)	No PCOS	PCOS	RR (95 % CI)	P value
20	$3709	$3954	1.07 (1.02–1.12)	0.006
30	$3971	$4377	1.10 (1.06–1.14)	<0.001
40	$4251	$4847	1.14 (1.09–1.20)	<0.001

Among the primary diagnoses of different mood disorders for females between 18 and 50 years of age, anxiety, mania, bipolar disorder, UMD, and PMD demonstrated no statistically significant variance between non-PCOS and PCOS groups (Table 3). Anxiety showed 5828 (2 %) and 78 (2 %) between the non-PCOS and PCOS groups, respectively (p = 0.653). Mania yielded 803 (<1 %) and 6 (<1 %) (p = 0.115). Bipolar disorder demonstrated 88,168 (37 %) and 1237 (37 %) (p = 0.878). PMD showed 1327 (1 %) and 24 (1 %) (p-value = 0.228). PAD demonstrated 173 (<1 %) and 1 (<1 %) (p = N/A). MDD and UMD were found to have statistically significant differences between the non-PCOS and PCOS; MDD showed 92,211 (39 %) and 1446 (43 %) (p=<0.001), suggesting a greater prevalence within the PCOS group; UMD showed 8024 (3 %) and 82 (2 %) (p = 0.005), suggesting a greater prevalence within the non-PCOS group.

**Table 3:** Primary diagnosis per ICD-10 codes used for patient characteristics in Table 4.

Diagnosis	No PCOS (N = 238,344)	PCOS (n = 3356)	P value
Anxiety	5828 (2 %)	78 (2 %)	0.653
Manic	803 (<1 %)	6 (<1 %)	0.115
Bipolar	88,168 (37 %)	1237 (37 %)	0.878
Depression	41,983 (18 %)	483 (14 %)	<0.001
MDD (Major Depressive Disorder)	92,211 (39 %)	1446 (43 %)	<0.001
UMD (Unspecified Mood Disorder)	8024 (3 %)	82 (2 %)	0.005
PMD (Persistent Mood Disorder)	1327 (1 %)	24 (1 %)	0.228
PAD (Panic Disorder)	173 (<1 %)	1 (<1 %)	N/A

In females aged 18–50 years old admitted with a mood disorder, the non-PCOS group had a median age of 32 and an interquartile range of 41 compared to the PCOS group with a median age of 28 and an interquartile range of 35 (P < 0.001) (Table 4).

**Table 4:** Demographic factors comparing PCOS and non-PCOS patients hospitalized with mood disorders.

Patient Characteristics	No PCOS (N = 238,344)	PCOS (n = 3356)	P value
**Median Age (IQR)**	32 (24, 41)	28 (23, 35)	<0.001
Race			<0.001
White	163,772 (69 %)	2578 (77 %)	
Black	37,968 (16 %)	359 (11 %)	
Hispanic	22,684 (10 %)	263 (8 %)	
Other	13,920 (6 %)	156 (4 %)	
**Insurance**			<0.001
Medicare	27,470 (12 %)	399 (12 %)	
Medicaid	95,239 (40 %)	1021 (30 %)	
Private	82,984 (35 %)	1585 (47 %)	
Other	32,651 (14 %)	351 (10 %)	
**Hospital Type**			0.103
Rural	25,024 (10 %)	345 (10 %)	
Urban Non-Teaching	54,012 (23 %)	703 (21 %)	
**Urban Teaching**	159,308 (67 %)	2308 (69 %)	
**Weekend Admission**	48,324 (20 %)	639 (19 %)	0.076
**Median Income (quartile by ZIP code)**			<0.001
Quartile 1	77,298 (33 %)	905 (27 %)	
Quartile 2	64,197 (28 %)	972 (29 %)	
Quartile 3	52,302 (22 %)	813 (25 %)	
Quartile 4	39,164 (17 %)	618 (19 %)	

In stratifying PCOS and no PCOS patients based upon racial group, 163,772 (69 %) females without PCOS admitted for a mood disorder were white compared to 2578 (77 %) with PCOS were white (P < 0.001) (Table 4). There were 37,968 (16 %) admitted black patients without PCOS compared to 359 (11 %) black patients with PCOS (P < 0.001). There were 22,684 (10 %) Hispanic patients without PCOS compared to 263 (8 %) Hispanic patients (P < 0.001). In addition, 13,920 (6 %) patients who were admitted for a mood disorder without PCOS identified as “other” for their racial category, compared to 156 (4 %) patients in the PCOS group (P < 0.001).

The data was further stratified based upon insurance type: Medicare, Medicaid, private insurance, or other. For women without a diagnosis of PCOS admitted for a mood disorder 27,470 (12 %) had Medicare, compared to 399 (12 %) with PCOS (P < 0.001) (Table 4). In the non-PCOS group, 95,239 (40 %) had Medicaid, compared to 1021 (30 %) with a concomitant diagnosis of PCOS (P < 0.001). There were 82,984 (35 %) admitted patients without PCOS who had private insurance, compared to 1585 (47 %) patients with PCOS (P < 0.001). 32, 651 (14 %) patients without PCOS were classified in the “other” insurance category, while 351 (10 %) of PCOS patients were classified into this category (P < 0.001).

Upon stratifying based on hospital type (rural, urban non-teaching, urban teaching), it was elucidated that 25,024 (10 %) patients admitted with a mood disorder without PCOS were treated at a rural hospital compared to 345 (10 %) patients with PCOS (P = 0.103) (Table 4). 54,012 (23 %) patients without PCOS were treated at an urban non-teaching hospital, compared to 703 (21 %) patients with PCOS who were treated at similar institution types (P = 0.103). There were 159,308 (67 %) patients without a diagnosis of PCOS treated at an urban teaching hospital, in comparison to 2308 (69 %) patients with PCOS treated at an urban teaching institution (P = 0.103).

In comparing patients admitted for a mood disorder, 48,324 (20 %) were admitted on a weekend day, compared to 639 (19 %) patients with a diagnosis of PCOS (P = 0.076) (Table 4).

The median income by ZIP was stratified into quartiles and subsequently analyzed (Table 4). Quartile 1 yielded 77,298 of 238,344 (33 %) of the non-PCOS group and 905 of 3356 (27 %) of the PCOS group. Quartile 2 yielded 64,197 (28 %) and 972 (29 %) of the non-PCOS and PCOS groups, respectively. Quartile 3 yielded 52,302 (22 %) and 813 (25 %) of the non-PCOS and PCOS groups, respectively. Quartile 4 yielded 39,164 (17 %) and 618 (19 %) of the non-PCOS and PCOS groups, respectively. In summary, the median income quartiles generated a p-value <0.001.

## 4 Discussion

### 4.1 Pathophysiology of PCOS and mood disorders

In the context of insulin resistance, elevated serum insulin levels increase ovarian androgen production and inhibit hepatic sex-hormone binding globulin (SHBG) production, thereby synergistically boosting free testosterone levels [12]. Through various intracellular pathways, excess androgen activity on its receptors generates a pathological metabolic profile in the nerves, liver, pancreatic beta cells, skeletal muscle, and fat consistent with obesity, diabetes mellitus, and worsening insulin resistance [13]. The resulting irregular menstrual cycles subsequently cause the manifestation of depression and anxiety [14].

Current literature is limited on the precise pathophysiological link between PCOS and mood disorders, but available data suggest a positive correlation of symptoms of depression with serum androgen levels, obesity, and degree of insulin resistance [6,15]. Neuronal regulation of both mood and appetite is possibly linked through the central melanocortin system involving pro-opiomelanocortins, agouti-related protein, neuropeptide Y, and melanocortin receptors which regulate both feeding and development of anxiety and depression [16]. In rats, stimulation of the melanocortin-4-receptor pathway induced hyperandrogenic phenotype similar to human females with PCOS [17]. Mood disorders also showed a direct relationship between the severity of mood symptoms and the severity of PCOS symptoms. The average Beck Depression Inventory (BDI) score, which standardizes depression symptom severity, was significantly higher with comorbid PCOS in comparison to otherwise healthy women [18].

### 4.2 Hospital cost

Between women with and without PCOS, hospital costs were significantly increased for the former, both unadjusted and adjusted for age, insurance, hospital type, admission date, and income. As women aged, the absolute difference of healthcare costs accumulated by women with PCOS and mood disorders also steadily increased compared to that of women with only mood disorders. One likely explanation for this phenomenon may be the increased baseline predilection of developing metabolic dysregulation with aging (i.e. weight gain, hypertension, and diabetes) [19]. Additional PCOS as a metabolic disorder creates a combination for a more complicated patient profile, leading to longer hospital stays and greater need for expensive interventions.

Of the $15 billion dollars spent on managing PCOS annually, the associated mental health disorders with the highest economic burdens are anxiety, depression, and eating disorders, collectively costing over $4 billion in the United States in 2021 [11]. The financial breakdown by Yadav, S. et al. showed that 28 % of the total annual cost was allocated to the treatment of mood disorders associated with PCOS, compared to 29.5 % for treating the actual endocrine derangements, 15.1 % for peri- and intrapartum complications, 10.1 % for comorbid type 2 diabetes mellitus, and 16.1 % for related strokes [11]. Additional analysis by Riestenberg, C. et al. calculated the cost of PCOS-associated type 2 diabetes mellitus, one of the most prominent comorbidities of PCOS, to be approximately $1.5 billion in 2020 [20]. In the greater context of female pathologies, the total healthcare costs of the most common female cancers are $29.8 billion for breast cancer, $23.8 billion for lung cancer, and $24.3 billion for colorectal cancer in 2020 [21]. Furthermore, the diagnostic cost of PCOS was $166 million in 2021, consisting of only 1.1 % of the total healthcare burden [11]. Considering that PCOS-related costs are almost half of that of breast cancer, of which the cost of mood disorders account for almost a third of the total cost, it would be an efficacious strategy to promptly reduce healthcare spending by addressing PCOS-related mood disorders and the prevention and early detection of PCOS via medical management options and policy changes, both of which may potentially be faster and cheaper to implement than cancer treatment development and implementation [22].

### 4.3 Length of stay

Likewise, inpatient duration accrued by women with PCOS and mood disorders was significantly longer than women hospitalized with mood disorders without PCOS. This increase may be similarly attributed to the need for more complex, time-intensive management than with either alone. In particular, retrospective studies done in England and using the NIS have demonstrated significantly longer hospital stays for patients with mood disorders than without, especially pronounced in Asian patients, although the exact treatment plans used were not disclosed [23,24]. Moreover, the tendency of PCOS extending into multiple physiological and psychosocial systems requires a multidimensional approach to care. Almhmoud, H. et al. delineates a management strategy involving the combination of pharmacotherapy, physical therapy, nutritional regimentation, sleep hygiene, cognitive behavioral therapy (CBT), counseling, and mindfulness-based stress reduction [25]. The proper execution of a comprehensive treatment plan such as the example mentioned requires extensive coordination among multiple healthcare disciplines, including but not limited to physicians, physical and occupational therapists, nutritionists, psychologists, mental health therapists, and social workers, which may likely equate to prolongation of the hospital course. Continued research into the efficacy of various management modalities for women with PCOS and related mood disorders would aid in the better understanding and optimization of care for the providers involved such that length of stay could be substantially shortened.

### 4.4 Demographic factors

Secondarily, there remains a large gap in current literature regarding the effect of demographic factors on the relationship between PCOS and mood disorders. Existing data suggest that African American and Hispanic women have greater propensity toward metabolic dysfunction, depression, and anhedonia than their Caucasian counterparts [26]. Of particular note, Asian women were also found to have increased risk of diabetes despite lower median obesity rates, although mood burden has not been documented. The greater population component of lower socioeconomic status has well been established as a prominent factor for increased risk of health-adverse behaviors (e.g. smoking, sedentary lifestyle, poor diet) that may be linked to obesity and development of PCOS [27,28].

Our data suggest the presence of PCOS has a significant impact on women with mood disorders to be hospitalized at a younger age. This is consistent with the observation that testosterone production wanes as the ovaries and adrenal glands age, decreasing the overall volume and output [29]. Therefore, younger women experience PCOS driven by hyperandrogenic features (e.g. hirsutism, acne). However, it is important to mention that PCOS in older women is largely attributed to greater insulin resistance associated with aging. Given that age of diagnosis occurs mostly in reproductive-age women (20–30s), we surmise hyperandrogenism is the predominant driving force and thus more prevalent in younger women [30].

Our findings for race and ethnicity sub-stratification demonstrated that Caucasians were the overall racial majority of patients with mood disorders regardless of PCOS status, although significantly more Caucasians had PCOS than without. Conversely, the minority of hospitalized women with mood disorders consisted of African Americans, Hispanics, and other ethnicities, and even fewer of whom had PCOS. It is important to note that these results oppose the general conclusions in current literature, with a specific exception being a cross-sectional study at the University of Pennsylvania between 2015 and 2018 by Alur-Gupta, S. et al. which found that Caucasians with PCOS had a significantly higher prevalence of anxiety than African Americans with PCOS, with no notable difference in depression among the two groups [31]. We hypothesize this dichotomy may be due to limitations of the NIS database imparting a degree of patient profile bias, differences in inclusion criteria and definitions, inadequate data collection of the endpoints, and/or changing demographic trends for PCOS in the past decade. In any case, the conflicting results should warrant the need for further analysis of the role of race in PCOS and concurrent mood disorders.

Briefly, our analysis demonstrated a majority of hospitalized women with mood disorders regardless of PCOS status under either Medicaid or private insurance. There was a minority (12 %) of patients in both groups who were insured by Medicare. By law, the age for Medicare eligibility begins at 65 years old, with earlier exceptions being individuals with disability, end-stage renal disease (ESRD), or amyotrophic lateral sclerosis (ALS) [32]. Mood disorders qualify for Social Security disability (SSDI) and Medicare coverage [33]. Considering our inclusion criteria are women between ages 18–50 years old, our findings suggest mood disorders cause significant incapacitation to living, including employment, such that a disproportionate number of women require SSDI and Medicare to survive. While this conclusion is more of a testament to the impact of mood disorders, it is nonetheless important to manage PCOS, which is a major comorbidity and a potential source of mood disorders.

Our results showed a greater proportion of women with PCOS covered by private insurance than Medicaid, which correlates with the upper income quartiles dominated by women with PCOS. We expected this finding given that individuals are more likely to switch from public to private health insurance as income increases [34]. However, as mentioned before, low socioeconomic status is strongly associated with metabolic dysfunction, a known pathological process underlying PCOS [27,28]. We suspect this phenomenon is likely due to the crucial importance of childhood socioeconomic status in the manifestation of metabolic dysfunction. Merkin, SS. et al. found a strong association between low childhood socioeconomic status and PCOS even in women with high incomes as adults [27]. In other words, poor lifestyle and education during childhood have a significantly greater weight in determining the risk of developing PCOS than any other point in a woman's lifespan.

### 4.5 Limitations

While our study takes advantage of the breadth and longitude of a public database, limitations are to be considered. It is possible our reliance on retrospective data carries a risk of data input error or misclassification. Outpatient interactions, observation-only hospital stays, and multiple hospitalizations by the same patient are not recorded. The limited time span of investigation between 2016 and 2020 may prohibit adequate capture of overarching trends and/or sufficient representation able to be appropriately extrapolated to the general U.S. female population. In addition, our patient sample restricted to women between the ages 18–50 years old precludes our ability to generalize our findings to senior women older than age of 65. We acknowledge our definition of mood disorders per diagnosis by ICD-10 codes may not completely scrutinize the nuances of specific mood disorders as conventionally described by the American Board of Psychiatry and Neurology (ABPN) and the DSM-5.

## Presentation

56th Annual Midwest Student Biomedical Research Forum (2025).

## CRediT authorship contribution statement

**Bob Weng:** Conceptualization, Data curation, Formal analysis, Investigation, Methodology, Resources, Writing – original draft, Writing – review & editing. **Reid Morrissey:** Conceptualization, Data curation, Formal analysis, Investigation, Methodology, Resources, Writing – original draft, Writing – review & editing. **Jenna Lehn:** Conceptualization, Data curation, Formal analysis, Investigation, Methodology, Resources, Writing – original draft, Writing – review & editing. **Mustafa Beidas:** Conceptualization, Data curation, Formal analysis, Investigation, Methodology, Resources, Writing – original draft, Writing – review & editing. **Tauseef Abubakar:** Supervision, Validation, Visualization. **Mohsin Mirza:** Supervision, Validation, Visualization.

## Assistance with the study

None.

## Financial support and sponsorship

None.

## Declaration of competing interest

The authors declare that they have no known competing financial interests or personal relationships that could have appeared to influence the work reported in this paper.
