# Supplementary material for: Influence of mood disorders on outcomes of polycystic ovarian syndrome - A national inpatient sample study - 2016–2020
Source: Compr Psychoneuroendocrinol. 2025 Jun 4;23:100305. doi: 10.1016/j.cpnec.2025.100305 (PMC12173662; doi:10.1016/j.cpnec.2025.100305)
Supplement: Multimedia component 1 [file mmc1.docx]

**Supplemental Files**

| **Mood Disorders** | **ICD-9** | **ICD-10** |
| --- | --- | --- |
| Depression (mild) | 296.21 | F32.0 |
| Depression (moderate) | 296.22 | F32.1 |
| Depression (severe) | 296.23 | F32.2 |
| Depression (severe+psychosis) | 296.24 | F32.3 |
| Recurrent Depressive Disorder (mild) | 296.31 | F33.0 |
| Recurrent Depressive Disorder (moderate) | 296.32 | F33.1 |
| Recurrent Depressive Disorder (severe) | 296.33 | F33.2 |
| Recurrent Depressive Disorder (severe+psychosis) | 296.34 | F33.3 |
| Seasonal Affective Disorder | 296.99 | F33.9 |
| Dysthymia | 300.4 | F34.1 |
| Generalized Anxiety Disorder | 300.02 | F41.1 |
| Adjustment Disorder | 309.28 | F43.20 |
| Panic Disorder | 300.01 | F41.0 |
| Anxiety State (unspecified) | 300.00 | F41.1 |
| Bipolar Disorder (current episode hypomanic) | 296.4 | F31.0 |
| Bipolar Disorder (current episode manic without psychosis) | 296.4 | F31.1 |
| Bipolar Disorder (current episode manic with psychosis) | 296.44 | F31.2 |
| Bipolar Disorder (current episode mild/moderate depression) | 296.5 | F31.3 |
| Bipolar Disorder (current episode severe depression without psychosis) | 296.53 | F31.4 |
| Bipolar Disorder (current episode severe depression with psychosis) | 296.54 | F31.5 |
| Bipolar Disorder (current episode mixed) | 296.6 | F31.6 |
| Bipolar Disorder (current remission) | 296.7 | F31.7 |
| Obsessive Compulsive Disorder | 300.3 | F42.9 |
| Somatization Disorder | 300.81 | F45.0 |

Supplemental Table 1: Respective ICD-9 and ICD-10 codes used for data extraction of mood disorders from the NIS.
